# Dietary resveratrol attenuation of intestinal inflammation and oxidative damage is linked to the alteration of gut microbiota and butyrate in piglets challenged with deoxynivalenol

**DOI:** 10.1186/s40104-021-00596-w

**Published:** 2021-06-16

**Authors:** Yueqin Qiu, Jun Yang, Li Wang, Xuefen Yang, Kaiguo Gao, Cui Zhu, Zongyong Jiang

**Affiliations:** 1 State Key Laboratory of Livestock and Poultry Breeding; Key Laboratory of Animal Nutrition and Feed Science in South China, Ministry of Agriculture and Rural Affairs; Guangdong Provincial Key Laboratory of Animal Breeding and Nutrition; Maoming Branch, Guangdong Laboratory for Lingnan Modern Agriculture, Institute of Animal Science, Guangdong Academy of Agricultural Sciences, Guangzhou, 510640 China; 2 College of Animal Science, South China Agricultural University, Guangzhou, 510642 China; 3 School of Life Science and Engineering, Foshan University, Foshan, 528225 China

**Keywords:** Deoxynivalenol, Gut health, Gut microbiota, Inflammation, Oxidative damage, Piglets, Resveratrol

## Abstract

**Background:**

Deoxynivalenol (DON) is a widespread mycotoxin that induces intestinal inflammation and oxidative stress in humans and animals. Resveratrol (RES) effectively exerts anti-inflammatory and antioxidant effects. However, the protective effects of RES on alleviating DON toxicity in piglets and the underlying mechanism remain unclear. Therefore, this study aimed to investigate the effect of RES on growth performance, gut health and the gut microbiota in DON-challenged piglets. A total of 64 weaned piglets [Duroc × (Landrace × Yorkshire), 21-d-old, 6.97 ± 0.10 kg body weight (BW)] were randomly allocated to 4 treatment groups (8 replicate pens per treatment, each pen containing 2 males; *n* = 16 per treatment) for 28 d. The piglets were fed a control diet (CON) or the CON diet supplemented with 300 mg RES/kg diet (RES group), 3.8 mg DON/kg diet (DON) or both (DON+RES) in a 2 × 2 factorial design.

**Results:**

DON-challenged piglets fed the RES-supplemented diet had significantly decreased D-lactate concentrations and tumor necrosis factor alpha (*TNF-α*) and interleukin 1 beta (*IL-1β*) mRNA and protein expression, and increased zonula occludens-1 (*ZO-1)* mRNA and protein expression compared with those of DON-challenged piglets fed the unsupplemented diet (*P *< 0.05). Compared with unsupplemented DON-challenged piglets, infected piglets fed a diet with RES showed significantly decreased malondialdehyde (MDA) levelsand increased mRNA expression of antioxidant enzymes and antioxidant genes (i.e., *GCLC, GCLM, HO-1, SOD1* and *NQO-1)* and glutamate-cysteine-ligase modulatory subunit (GCLM) protein expression (*P* < 0.05). Moreover, RES supplementation significantly abrogated the increase in the proportion of TUNEL-positive cells and the protein expression of caspase3 in DON-challenged piglets (*P *< 0.05). Finally, RES supplementation significantly increased the abundance of *Roseburia* and butyrate concentrations, while decreasing the abundances of *Bacteroides* and *unidentified-Enterobacteriaceae* in DON-challenged piglets compared with DON-challenged piglets alone (*P *< 0.05).

**Conclusions:**

RES supplementation improved gut health in DON-challenged piglets by strengthening intestinal barrier function, alleviating intestinal inflammation and oxidative damage, and positively modulating the gut microbiota. The protective effects of RES on gut health may be linked to increased *Roseburia* and butyrate concentrations, and decreased levels of *Bacteroides* and *unidentified-Enterobacteriaceae*.

## Background

Deoxynivalenol (DON), also known as vomitoxin, is a major mycotoxin detected in food crops and livestock feed [1]. Due to its toxic properties and high stability, the presence of DON in the food chain increases the risks to human health and animal productivity [2]. Increasing evidence has indicated that ingestion and absorption of DON-contaminated feed causes a series of toxic symptoms in both humans and animals, such as vomiting, diarrhea, feed refusal, inflammation, and reduced weight gain [3, 4]. Among domestic animals, pigs exhibit increased sensitivity to DON [5]. The intestinal barrier is the first line of defense against encroaching commensal bacteria, invading enteric pathogens and natural toxins [6]. Previous studies have reported that DON exposure induces inflammatory responses, intestinal permeability and enterocyte apoptosis, which contribute to disruption of the intestinal barrier and immune function in piglets [1, 7, 8]. Moreover, it has been reported that DON also induces oxidative damage in intestinal cells [9–11]. Therefore, preventing DON-induced intestinal oxidative stress imbalance and inflammation may be a potential strategy to treat intestinal injury.

In the recent decade, phenolic compounds extracted from plants have gained a tremendous amount of significant attention due to their diverse health-promoting properties in swine production. Numerous studies have shown that polyphenols are capable of alleviating intestinal disorders associated with oxidative stress and inflammation, inhibiting pathogenic microorganisms, promoting epithelial integrity and improving the growth performance and feed efficiency of weanling piglets [12–17]. Among polyphenols, resveratrol (RES), which is principally found in grapes, red wine, and berries [18], effectively exerts anti-inflammatory and antioxidant effects and improves the growth performance of weaned piglets [12, 14, 19, 20]. In animal models, RES has been shown to improve the intestinal barrier and immune functions [12, 21, 22]. Likewise, cellular studies have demonstrated that RES protects against oxidative damage and intestinal inflammation by activating nuclear factor E2-related factor 2 (NRF2) and inhibiting nuclear factor kappa B (NF-κB) [11, 23]. However, the protective effects of RES on the inhibition of DON toxicity in piglets remain unclear.

The gut microbiota plays an important role in suppressing pathogen infection and regulating nutrient digestion and absorption and closely shapes intestinal barrier function [24]. Although numerous studies have shown a deleterious effect of DON exposure on the gastrointestinal tract, only a few studies have investigated the effect of DON on the intestinal microbial ecosystem of piglets [25, 26]. Furthermore, whether DON induces intestinal damage and growth inhibition in piglets by altering the gut microbiota remains unclear. It has been reported that polyphenols contribute to gut health via modulation of the colon microbiota composition. Several studies demonstrated that supplementation with polyphenols changed the composition of the microbiome favoring the growth of beneficial bacteria while inhibiting the growth of pathogenic bacteria in pigs and other animal models [12, 16, 17, 27, 28]. A previous study from Meng et al. reported that dietary resveratrol increased the proportion of butyrate-producing bacteria in weaned piglets [12]. However, the mechanism by which RES exerts its protective effect on the intestine by altering gut microbiota composition in DON-challenged piglets remains unclear.

Hence, in the present study, we hypothesized that RES alleviated intestinal damage and improved growth performance in DON-challenged piglets by altering the gut microbiota. The effects of RES supplementation on the growth performance, intestinal barrier integrity, intestinal oxidative stress, gut inflammation and gut microbiota of weaned piglets challenged with DON were investigated.

## Materials and methods

All animal procedures used in this study were approved by the Animal Care and Use Committee of Guangdong Academy of Agricultural Sciences (authorization number GAASIAS-2016-017). All efforts were made to minimize animal suffering in accordance with the Guidelines for the Care and Use of Animals for Research and Teaching [29].

### Preparation of DON-contaminated feed

*Fusarium graminearum* strain R6576, which is only able to produce DON, was kindly provided by the College of Plant Science & Technology of Huazhong Agricultural University, China. DON-contaminated corn was prepared according to previous studies [30]. The moldy corn was determined to contain 7.80 mg DON/kg corn. Before the treatments, moldy corn was mixed with a basal diet to provide 3.8 mg DON/kg diet for the DON and DON+RES treatments.

### Animals and diets

A total of 64 weaned piglets [Duroc × (Landrace × Yorkshire), 21 days old, barrow] with an initial weaning weight of 6.97 ± 0.10 kg were randomly allocated to 4 dietary treatments. The piglets fed a basal diet were considered the control group (CON), and the other groups were fed the basal diet supplemented with 300 mg RES/kg diet (RES), 3.8 mg DON/kg diet (DON) or 3.8 mg DON plus 300 mg RES per kg diet (DON+RES group) for a 28-days feeding trial. RES (> 99.0%) was obtained commercially from Shanxi Ciyuan Biotechnology Co., Ltd. (Xian, China). Each treatment consisted of 8 replicate pens, with 2 piglets per pen (*n* = 16 piglets per treatment). The basal diet was formulated to meet the nutrient recommendations of the National Research Council (NRC) 2012 [31]. Diet compositions and nutrient profiles are presented in Table 1. All pigs had free access to feed and water during the entire feeding period. At the end of the study, the average daily gain (ADG), feed intake and gain/feed were calculated. One pig from each pen was randomly selected, anesthetized with sodium pentobarbital (40 mg/kg body weight (BW)) and sacrificed. The entire intestine was rapidly removed and placed on a cold tray to collect the jejunum. Two continuous segments were carefully cut from the middle of the whole jejunum for histological assay, intestinal mitochondria ultrastructure and mucosa collection. Sections of approximately 2.0 cm in length were rinsed with ice-cold PBS and fixed in 4% paraformaldehyde for morphometric evaluation and histochemical staining. Additionally, approximately 0.5 cm × 0.5 cm jejunal segments were collected and fixed in 2.5% glutaraldehyde to analyze the intestinal mitochondrial ultrastructure. Approximately 20 cm portions of the jejunum were opened longitudinally and cleaned with ice-cold phosphate buffer solution (PBS). Mucosa samples were collected by scraping with sterile glass microscope slides, snap-frozen in liquid nitrogen and stored at − 80 °C for subsequent analysis. The colon digesta were also immediately collected, frozen in liquid nitrogen and stored at − 80 °C for gut microbiota and short-chain fatty acid (SCFA) analysis.

**Table 1:** Ingredient composition and nutrient levels of the basal diet (%, as-fed basis)

Item	
Ingredient composition, %	
Corn	34.00
Expanded corn	14.72
Soybean meal	10.00
Expanded soybean	8.50
Fishmeal	4.00
Low protein whey powder	11.00
Soybean hull	5.00
Plasma protein powder	4.00
Soybean oil	1.35
Sucrose	2.00
CaHPO_4_	1.20
Limestone powder	0.65
NaCl	0.45
Choline chloride 50%	0.20
L-Lysine HCl	0.82
DL-Methionine	0.25
L-Threonine	0.30
Trp	0.06
Premix^a^	1.50
Total	100
Nutrient levels^b^	
DE, kcal/kg	3516.62
CP, %	19.26
SID Lys, %	1.55
SID Met+Cys, %	0.78
SID Thr, %	0.88
SID Trp, %	0.25

^a^Supplied per kilogram of complete diet: acidifier 4 g, artificial sweetener 0.2 g, feed flavor 1 g, phytase 0.2 g, compound enzyme preparation 4 g, ZnO 2 g, zeolite powder 1 g, vitamin A 12,400 IU, vitamin D_3_ 2,800 IU, vitamin E 130 mg, vitamin K 5 mg, vitamin B_1_ 3 mg, vitamin B_2_ 10 mg, vitamin B_3_ 40 mg, vitamin B_5_ 15 mg, vitamin B_6_ 8 mg, vitamin B_12_ 40 μg, folic acid 1 mg, biotin 0.08 mg, Fe (FeSO_4_·H_2_O) 120 mg, Cu (CuSO_4_·5H_2_O) 16 mg, Mn (MnSO_4_·H_2_O) 70 mg, Zn (ZnSO_4_·H_2_O) 120 mg, I (CaI_2_O_6_) 0.7 mg, Se (Na_2_SeO_3_) 0.48 mg

^b^Nutrient levels are calculated based on the NRC (2012) database and Tables of feed composition and nutritive values in China (the values of sucrose are from Tables of feed composition and nutritive values in China, and others are from NRC (2012)). *CP* Crude protein, *DE* Digestible energy, *SID* Standardized ileal digestibility

### Intestinal morphology analysis

The fixed samples were embedded in paraffin, and 4-μm cross sections from each specimen were mounted on slides coated with polylysine, deparaffinized, rehydrated, and then stained with hematoxylin-eosin (HE) for jejunal morphological examination, and periodic acid-Schiff for the determination of goblet cells. HE-stained slices were scanned using a digital brightfield microscope scanner (Pannoramic 250, 3D HISTECH, Hungary). Twenty-five well-oriented and intact villi and adjacent crypts were randomly selected to measure the villus height and crypt depth of each segment using slide viewer software (Case Viewer 2.3, 3D HISTECH, Hungary), and the villus height-to-crypt depth ratio (VCR) was calculated. The number of goblet cells was counted based on the length and width of the goblet cell “cup” in cross-sections of the villi under a Zeiss light microscope (Zeiss, Germany). The density of goblet cells was calculated as the number of goblet cells per unit surface area (mm^2^).

### Ultrastructure of intestinal mitochondria

Jejunal segments were fixed in 2.5% glutaraldehyde for 24 h. The samples were dehydrated in gradient concentrations of ethanol and embedded in Spurr resin, after which ultrathin sections were stained with uranyl acetate and alkaline lead citrate for 5 to 10 min, placed in Eppendorf tubes containing Spurr resin and heated at 70 °C for 12 h. The specimens were then sectioned using a LEICA EM UC7 ultratome (Leica, Weztlar, Germany) and stained with uranyl acetate and alkaline lead citrate for 5 and 10 min, respectively. Finally, the sections were observed using a Hitachi Model H-7650 transmission electron microscope (TEM) (Hitachi, Tokyo, Japan).

### Enzyme-linked immunosorbent assay (ELISA)

The concentrations of interleukin 1 beta (IL-1β), interleukin 6 (IL-6) and tumor necrosis factor alpha (TNF-α) in the jejunal mucosa were determined using commercial ELISA kits (Cusabio Biotech Co., Ltd., Wuhan, China) according to the manufacturer’s instructions. Before assays, approximately 0.1 g frozen jejunal mucosa was homogenized with ice-cold saline (1:9, w/v) for 2 min. The homogenates were centrifuged (5,000×*g* for 10 min at 4 °C). Then the supernatant was collected and rapidly used to determine the cytokine levels. Cytokine content was standardized to the total protein in each sample.

The total protein concentrations of jejunal mucosa were measured by a BCA protein assay kit (Pierce, Rockford, IL, USA) with BSA standards.

### Analyses of intestinal antioxidant/oxidant indices

Approximately 0.1 g frozen jejunal mucosa was homogenized with ice-cold saline (1:9, w/v) for 2 min. The homogenates were then centrifuged (3,500×g for 15 min at 4 °C), and the supernatant was collected for intestinal antioxidant/oxidantindex analysis. Total antioxidant capacity (T-AOC), total superoxide dismutase (T-SOD) and the levels of glutathione (GSH) and MDA in the jejunal mucosa were determined using assay kits in accordance with the manufacturer’s protocol (Nanjing Jiancheng Institute of Bioengineering and Technology Nanjing, China).

### Determination of plasma diamine oxidase (DAO) activity and D-lactate concentrations

The plasma concentrations of DAO and D-lactate were measured using assay kits according to the manufacturer’s instructions (BioVision, Inc. San Francisco, USA).

### Immunohistochemistry for terminal deoxynucleotidyl transferase mediated dUTP nick end labeling (TUNEL)

The fixed samples were embedded in paraffin, and a 4-μm section was mounted on glass slides and then dried for 12 h at 37 °C. The prepared samples were passed through xylene and ingredient ethanol solutions for deparaffinization, and then immunohistochemistry stained using a TUNEL kit (Boster, Wuhan, China), which is a marker of apoptosis, in accordance with the manufacturer’s protocol.

### Quantitative real-time PCR (qPCR)

TRIzol reagent (Invitrogen, Carlsbad, CA) was used to isolate total RNA from jejunal mucosal samples in accordance with the manufacturer’s instructions. The purity and concentration of the RNA were determined using a NanoDrop-ND1000 spectrophotometer (Thermo Fisher Scientific Inc., Walldorf, Germany). One microgram of total RNA was used to synthesize cDNA using a PrimeScript™II 1st Strand cDNA Synthesis Kit (Takara, Tokyo, Japan). SYBR green I (Takara), 10-fold diluted cDNA and gene-specific primers (Table 2) in a final volume of 20 μL were used to perform qPCR analyses in triplicate. The qPCR conditions were 95 °C 3 min followed by 40 cycles of amplification (95 °C 15 s, 60 °C 30 s, and 72 °C 30 s). The beta actin (*ACTB)* gene was used as an internal control because its abundance was not significantly influenced by treatment in the present study (results not shown). The fold changes in target gene expression in piglets fed treatment diets were normalized to *ACTB* and relative to the expression in piglets fed the CON diet; fold changes were calculated for each sample using the 2^−ΔΔ*C*t^ method, where ΔΔ*C*_T_ = (*C*_T, Target_ - *C*_T, *ACTB*_)_Treatment_ - (Average *C*_T, Target_ - Average *C*_T, *ACTB*_)_Control_.

**Table 2:** Primer sequences used in this study

Genes	Sequences (5′→3′)		Product Size, bp	GenBank Accession
*Claudin-1*	Forward Reverse	ACGGCCCAGGCCATCTAC TGCCGGGTCCGGTAGATG	221	AJ318102.1
*ZO-1*	Forward Reverse	AGCCCGAGGCGTGTTT GGTGGGAGGATGCTGTTG	147	XM_021098827.1
*TNF-α*	Forward Reverse	CACGCTCTTCTGCCTACTGC GTCCCTCGGCTTTGACATT	164	NM_214022.1
*ACTB*	Forward Reverse	CATCGTCCACCGCAAAT TGTCACCTTCACCGTTCC	210	NC_010445
*Occludin*	Forward Reverse	GCACCCAGCAACGACAT CATAGACAGAATCCGAATCAC	144	XM_005672525
*SOD1*	Forward Reverse	GAGACCTGGGCAATGTGACT CTGCCCAAGTCATCTGGTTT	139	NM_001190422.1
*GCLC*	Forward Reverse	CAAACCATCCTACCCTTTGG ATTGTGCAGAGAGCCTGGTT	172	XM_003482164.4
*GCLM*	Forward Reverse	GATGCCGCCCGATTTAACTG ACAATGACCGAGTACCGCAG	177	XM_001926378.4
*HO-1*	Forward Reverse	CGCTCCCGAATGAACACTCT GCGAGGGTCTCTGGTCCTTA	148	NM_001004027.1
*NQO-1*	Forward Reverse	ATCACAGGTAAACTGAAGGACCC TGGCAGCGTATGTGTAAGCA	229	NM_001159613.1
*IL-1β*	Forward Reverse	CTCCAGCCAGTCTTCATTGTTC TGCCTGATGCTCTTGTTCCA	230	NM_214055.1
*IL-6*	Forward Reverse	TACATCCTCGGCAAAATC TCTCATCAAGCAGGTCTCC	168	NM_001252429.1
*IL-8*	Forward Reverse	AGGACCAGAGCCAGGAA GTGGAATGCGTATTTATGC	172	NM_213867.1
*TGF-β*	Forward Reverse	GAAGCGCATCGAGGCCATTC GGCTCCGGTTCGACACTTTC	162	NM_214015.2

*ACTB* Actin beta, *IL-1β* Interleukin 1 beta, *IL-6* Interleukin 6, *IL-8* Interleukin 8, *GCLC* Glutamate-cysteine-ligase catalytic subunit, *GCLM* Glutamate-cysteine-ligase modulatory subunit, *GSH* Glutathione, *HO*-1 Heme oxygenase-1, *NQO-1* NAD(P)H dehydrogenase, quinone 1, *SOD1* Superoxide dismutase 1, *TGF-β* Transforming growth factor beta, *TNF-α* Tumor necrosis factor alpha

### Western blot analysis

For each sample, approximately 0.5 g of frozen jejunum was lysed in 1 mL of RIPA buffer containing 1% protease inhibitor cocktail and 1% phosphatase inhibitor at 4 °C for 30 min and then centrifuged at 12,000×*g* at 4 °C for 15 min. The protein concentration of the extract was measured by a BCA protein assay kit (Pierce, Rockford, IL, USA) using BSA standards. Equal quantities of protein were diluted with 5× loading buffer, denatured at 100 °C for 10 min, cooled on ice, and then used for Western blot analysis. Denatured proteins were first separated by 8–10% SDS-PAGE and then transferred to nitrocellulose membranes. After being blocked with 5% BSA in TBST buffer for 30 min, the membranes were washed 4 times and then incubated with diluted primary antibodies at 4 °C overnight. After 4 washes, the membranes were incubated with the appropriate HRP-labeled secondary antibodies for 1 h at room temperature. After another 4 washes, we visualized immunoreactive proteins using the Immobilon Western Chemiluminescent HRP Substrate (Millipore, Billerica, MA, USA) and the VersaDoc Imaging System (Bio-Rad, Hercules, CA, USA), and ImageJ software (National Institutes of Health, Bethesda, MD, USA) was used to quantify the protein band intensities. Primary antibodies against zonula occludens-1 (ZO-1), occludin, claudin-1, claudin-3, superoxide dimutase 1 (SOD1), glutamate-cysteine-ligase catalytic subunit (GCLC), GCLM, B-cell lymphoma/leukemia 2 (Bcl-2), Bcl-2 associated x protein (Bax) and caspase-3 were purchased from Cell Signaling Technology (Boston, MA, USA). The results are expressed as the abundance of each target protein relative to β-actin.

### Gut microbiome analysis

Total DNA from each colonic digesta sample was extracted using the QIAamp PowerFecal DNA Kit (Qiagen, Hilden, Germany) according to the manufacturer’s instructions. The DNA concentration and quality of each sample were determined using a Nanodrop 2000 spectrophotometer (Thermo Fisher Scientific, Wilmington, DE, USA). All bacterial 16S rRNA genes covering the V3-V4 region were amplified with the universal forward primer 338F (5'-ACTCCTRCGGGAGGCAGCAG-3') and the reverse primer 806R (5'-GGACTACCVGGGTATCTAAT-3'). PCR amplicons were purified using the Qiagen Gel Extraction Kit (Qiagen, Germany) in accordance with the manufacturer’s instructions. Sequencing libraries were generated using a TruSeq® DNA PCR-Free sample preparation kit (Illumina, USA) according to the manufacturer’s recommendations, and the index codes were added. The quality of the library was then evaluated by a Qubit@ 2.0 fluorometer (Thermo Fisher Scientific, Carlsbad, CA, USA) and an Agilent Bioanalyzer 2100 system. The library was sequenced on an Illumina NovaSeq platform, and 250-bp paired-end reads were generated. The raw sequence data generated from 16S rRNA MiSeq sequencing were analyzed using Quantitative Insights into Microbial Ecology (QIIME, version 1.17). Gaps in each sequence were discarded from all samples to decrease noise by screening, filtering, and pre-clustering processes. The sequences were clustered into Operational taxonomic units (OTUs) with a cut-off of value 97% similarity by Uparse software (version 7.1, http://drive5.com/uparse/), and chimeric sequences were identified and removed using UCHIME algorithm. Mothur and SILVA132(http://www.arb-silva.de/)classifier tool were used to classify all sequences into different taxonomic groups. Bray-Curtis distance-based Principal Coordinate Analysis (PCoA) was performed to show the distribution of samples.

### Determination of SCFA concentrations

Colon content samples from weaned piglets were tested for the concentrations of acetate, propionate, and butyrate by LC using an LCMS-Xevo TQ-S Micro Ultra instrument (Waters, USA). Briefly, 0.05 g colon digesta from each sample was diluted with 1 mL 50% methanol and vortexed for 30 min until the mixture was homogenized. Then the homogenate was centrifuged at 12,000×*g* for 10 min at 4 °C. Fifty microliters of supernatant was collected, and 50 μL of internal standard and 100 μL of derivatization reagent were added for 30 min of derivation at room temperature. Then 750 μL of ultrapure water was added. Subsequently, this mixture was centrifuged at 12,000×*g* for 10 min at 4 °C. The collected supernatant was filtered through a 0.22-mm membrane and then analyzed using LC.

### Statistical analysis

The data were analyzed as a 2 × 2 factorial using PROC MIXED in SAS 9.3 (SAS Institute Inc., Cary, NC, USA). The model included DON (0 or 3.8 mg/kg diet), RES (0 or 300 mg/kg diet), and the interactive effects of DON and RES as the fixed effects, with pig identification as the random effects. The pen was the experimental unit for all analyses. The data are presented as the means and pooled SEM. Differences were considered significant if *P* ≤ 0.05 and to reflect a tendency if 0.05 < *P* < 0.10. When significant interactive effects occurred (*P* ≤ 0.05), post hoc testing was conducted using Duncan’s multiple comparison tests, and differences were considered significant when *P* ≤ 0.05. Correlations between the gut microbiota and gene and protein expression were analyzed by Pearson’s correlation using GraphPad Prism version 8.0 (GraphPad Software, San Diego, CA, USA), and significant differences were determined at *P ≤* 0.05.

## Results

### Effect of resveratrol supplementation on the growth performance of piglets challenged with deoxynivalenol

As shown in Table 3, no significant DON × RES interaction for BW gain or ADG was observed, but a tendency for a DON × RES interaction for gain/feed was observed. DON-challenged piglets had significantly lower BW gain (*P* < 0.001), ADG (*P* < 0.001), average daily feed intake (ADFI) (*P* < 0.01) and gain/feed (*P* < 0.001) than nonchallenged piglets. However, RES supplementation significantly increased BW gain (*P* < 0.01), ADG (*P* < 0.01) and gain/feed (*P* < 0.01) compared with diets without RES.

**Table 3:** Effects of resveratrol on growth performance in weaned piglets challenged with deoxynivalenol^a^

Variable	Treatments				SEM	*P-*value		
	CON	DON	RES	DON+RES		DON	RES	DON×RES
Initial BW, kg	6.97	6.98	6.97	6.98	0.03	0.70	0.85	0.96
Final BW, kg	14.21	12.69	14.87	13.63	0.23	< 0.001	0.002	0.53
ADG, g/d	258.75	203.82	282.28	237.66	8.08	< 0.001	0.002	0.53
ADFI, kg/d	410.92	369.27	435.91	390.63	12.57	0.002	0.076	0.89
Gain/Feed	0.63	0.54	0.65	0.61	0.01	< 0.001	0.002	0.06

^a^Values are means and standard error of the means, *n* = 8 per treatment; *ADG* Average daily gain, *ADFI* Average daily feed intake, *BW* Body weight, *CON* Control, *DON* Deoxynivalenol, *DON+RES* Combination of deoxynivalenol and resveratrol, *SEM* Standard error of the mean

### Effect of resveratrol supplementation on intestinal morphology and goblet cells in piglets challenged with deoxynivalenol

**Fig. 1:**
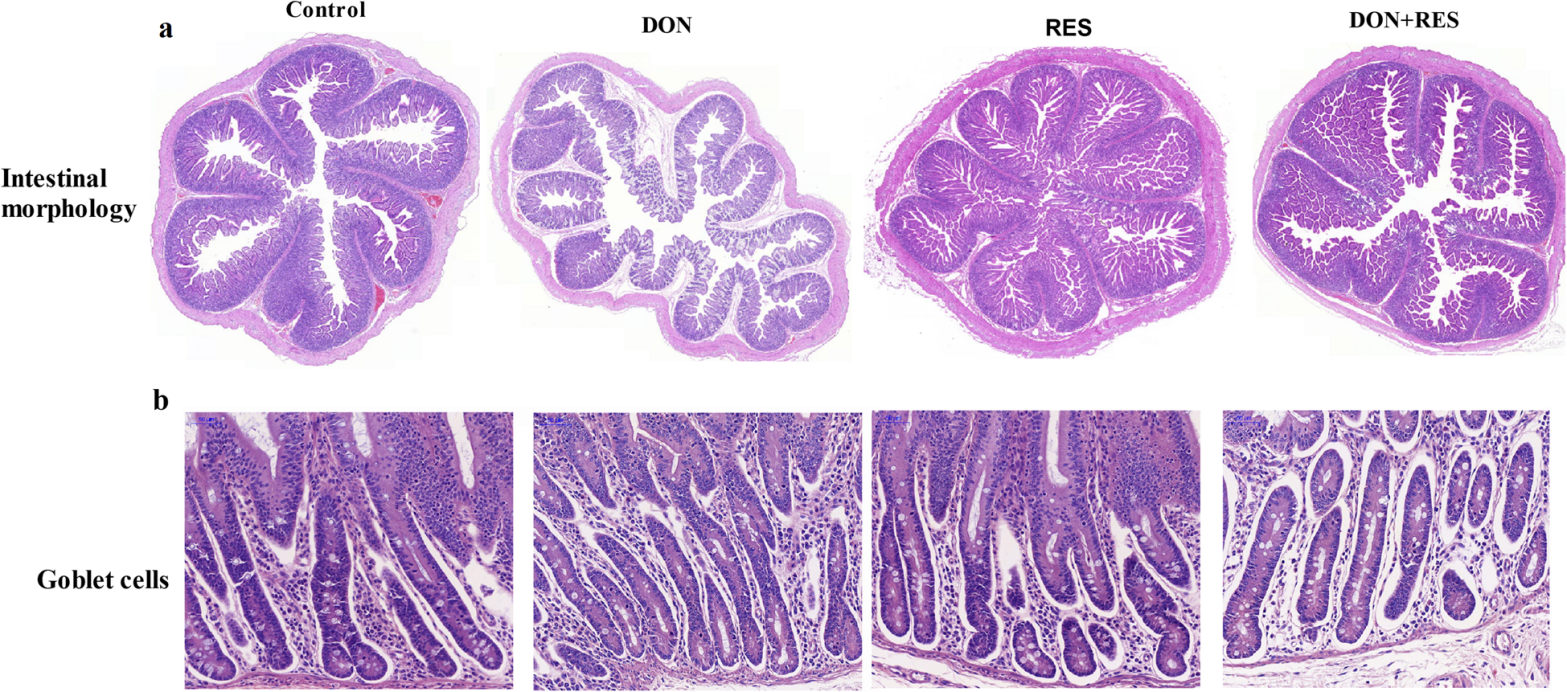
Effect of resveratrol supplementation on intestinal morphology and goblet cells in DON-challenged piglets. **a** Hematoxylin-eosin (H&E)-stained whole slide images. **b** The number of goblet cells in jejunal villus. DON, deoxynivalenol; DON+RES, combination of deoxynivalenol and resveratrol; RES, resveratrol; VCR, villus height to crypt depth; Note: Original magnification 150×, scale bar 50 μm

Histological analysis (Fig. 1 and Table 4) showed that DON exposure induced intestinal mucosal injury in the jejunum, as reflected by a shortened villus height (*P* = 0.001), reduced VCR (*P* = 0.001) and reduced numbers of goblet cells (*P* = 0.001) compared with the effects of diets lacking DON. RES supplementation significantly increased villus height (*P* = 0.001), VCR (*P* = 0.001) and the number of goblet cells (*P* = 0.003) and tended to decrease crypt depth (*P* = 0.055) compared with the effects of diets without RES. However, no significant DON × RES interaction for these variables was observed.

**Table 4:** Effect of resveratrol supplementation on intestinal morphology and goblet cells in DON-challenged piglets^a^

Variable	Treatments				SEM	*P*-value		
	CON	DON	RES	DON+RES		DON	RES	DON×RES
Villus height, μm	469	344	584	429	19	< 0.001	< 0.001	0.452
crypt depth, μm	346	354	306	336	14	0.186	0.055	0.468
VCR	1.38	0.98	1.92	1.29	0.09	< 0.001	< 0.001	0.202
Goblet cells^b^	29.13	19.25	33.56	25.13	1.57	< 0.001	0.003	0.650

^a^Values are means and standard error of the means, *n* = 8 per treatment; CON, control; DON, deoxynivalenol; DON+RES, combination of deoxynivalenol and resveratrol; SEM, standard error of the mean; VCR, villus height to crypt depth ratio

^b^Goblet cell numbers per mm^2^

### Effect of resveratrol supplementation on the activities of plasma DAO and D-lactate in piglets challenged with deoxynivalenol

**Fig. 2:**
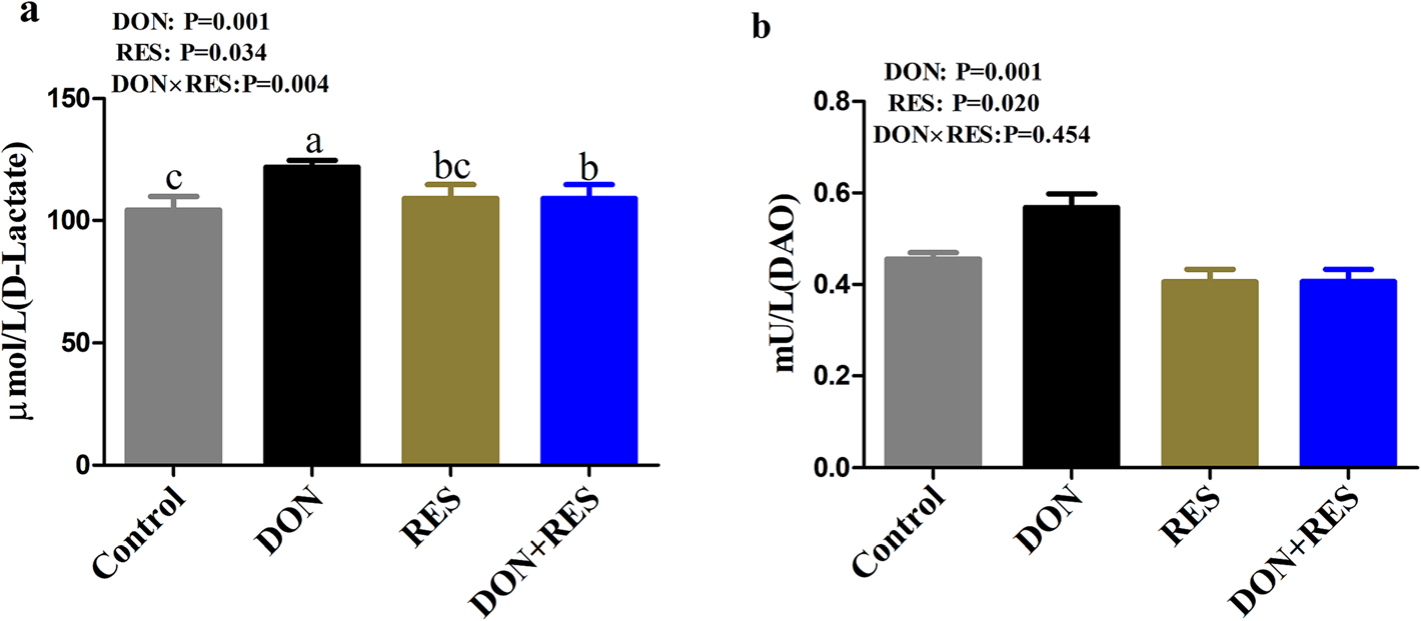
Effect of resveratrol supplementation on activities of plasma DAO and D-lactate in DON-challenged piglets. a DAO activity. b D-lactate level in the plasma of piglets. Data are presented as means ± SEM, *n* = 8; labeled means in a row without a common letter differ, *P* ≤ 0.05; DAO, diamine oxidase; DON, deoxynivalenol; DON+RES, combination of deoxynivalenol and resveratrol; RES, resveratrol

DON-challenged piglets had significantly higher plasma DAO (*P* = 0.001) and D-lactate (*P* = 0.001) concentrations than nonchallenged piglets (Fig. 2a and b). Compared with diets without RES, RES supplementation significantly decreased the concentrations of DAO (*P* = 0.034) and D-lactate (*P* = 0.020). A significant DON × RES interaction was observed for plasma D-lactate activity (*P* = 0.004), in which piglets administered a fed diet supplemented with DON and RES had lower plasma D-lactate activity than DON-challenged piglets alone (Fig. 2a and b).

### Effect of resveratrol supplementation on intestinal function and immunity in piglets challenged with deoxynivalenol

**Fig. 3:**
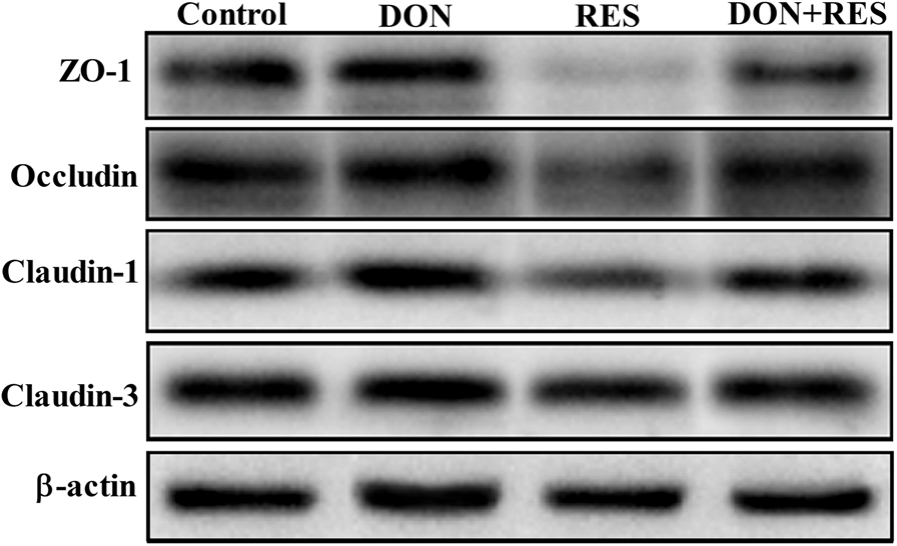
Effect of resveratrol supplementation on the intestinal function and immunity in DON-challenged piglets. Western blots analysis for the amounts of ZO-1, occludin, claudin-1 and claudin-3; loading and transfer of equal amounts of protein was confirmed by the immunodetection of β-actin. Similar results were obtained from 8 independent experiments. DON, deoxynivalenol; DON+RES, combination of deoxynivalenol and resveratrol; RES, resveratrol; ZO-1, zonula occludens-1

DON-challenged piglets had significantly lower mRNA expression levels of *ZO-1* (*P* = 0.001), occludin (*P* = 0.001), claudin-1 (*P* = 0.001) and transforming growth factor β (*TGF-β)* (*P* = 0.001) but higher mRNA levels of tumor necrosis factor α (*TNF-α)* (*P* = 0.001), interlleukin 6 *(IL-6)* (*P* = 0.001), interlleukin 1 beta *(IL-1β)* (*P* = 0.001) and interlleukin 6 *(IL-6)* (*P* = 0.011) in the jejunum than nonchallenged control piglets (Table 5). RES supplementation significantly increased claudin-1(*P* = 0.040), *ZO-1* (*P* = 0.001), occludin (*P* = 0.006) and *TGF-β* (*P* = 0.004) mRNA expression while decreasing the abundance of *TNF-α* (*P* = 0.002)*, IL-6* (*P* = 0.014) and *IL-1β* (*P* = 0.001) transcripts in the jejunum compared with the effects of diets without RES (Table 5). Additionally, a significant DON × RES interaction was identified with respect to the mRNA expression of *ZO-1* (*P* = 0.004)*, TNF-α* (*P* = 0.020) and *IL-1β* (*P* = 0.001); RES supplementation in DON-challenged piglets significantly reversed the DON-induced reduction in the mRNA expression of *ZO-1* and increases in *TNF-α* and *IL-1β* (Table 5). At the protein level, DON significantly decreased the protein levels of ZO-1 (*P* = 0.001), occludin (*P* = 0.001), claudin-1 (*P* = 0.001) and claudin-3 (*P* = 0.001) while increasing TNF-α (*P* = 0.001), IL-6 (*P* = 0.001) and IL-1β (*P* = 0.001) compared with the effects of diets without DON (Fig. 3 and Table 5). RES supplementation significantly increased the protein levels of ZO-1 (*P* = 0.001), occludin (*P* = 0.012), claudin-1 (*P* = 0.001) and claudin-3 (*P* = 0.006) while decreasing TNF-α (*P* = 0.001), IL-6 (*P* = 0.040) and IL-1β (*P* = 0.001) compared with the effects of diets without RES (Fig. 3 and Table 5). A significant DON ×RES interaction was observed for the protein levels of ZO-1 (*P* = 0.018), TNF-α (*P* = 0.026) and IL-1β (*P* = 0.041); DON-challenged piglets fed a diet supplemented with RES exhibited significantly increased ZO-1 protein levels and decreased protein levels of TNF-α and IL-1β compared with piglets challenged with DON alone (Fig. 3 and Table 5).

**Table 5:** Effects of resveratrol on the expression of genes and proteins related to tight junctions and immunity in the jejunal mucosa of weaned piglets challenged with deoxynivalenol ^a^

Variable	Treatments^2^				SEM	*P*-value		
	CON	DON	RES	DON+RES		DON	RES	RES × DON
Gene expressions								
*Claudin-1*	1.01^ab^	0.69^c^	1.18^a^	0.84^bc^	0.07	< 0.001	0.040	0.886
*ZO-1*	1.03^b^	0.52^c^	1.96^a^	0.90^b^	0.09	< 0.001	< 0.001	0.004
*Occludin*	1.02^b^	0.62^c^	1.41^a^	0.98^b^	0.12	0.005	0.002	0.936
*TNF-α*	1.03^bc^	2.08^a^	0.92^c^	1.37^b^	0.12	< 0.001	0.002	0.019
*TGF-β*	1.01^b^	0.32^d^	1.19^a^	0.51^c^	0.06	< 0.001	0.004	0.941
*IL-1β*	1.02^c^	3.30^a^	0.91^c^	1.86^b^	0.15	< 0.001	< 0.001	< 0.001
*IL-6*	1.03^c^	1.76^a^	0.86^c^	1.44^b^	0.09	< 0.001	0.014	0.386
*IL-8*	1.02^ab^	1.24^a^	0.93^b^	1.13^ab^	0.08	0.011	0.198	0.875
The cytokines levels, ng/g protein								
IL-1β	41.35^c^	69.45^a^	36.45^c^	52.68^b^	2.77	< 0.001	< 0.001	0.041
IL-6	45.19^b^	58.77^a^	40.67^b^	53.60^a^	2.25	< 0.001	0.040	0.888
TNF-α	7.59^c^	11.44^a^	6.94^c^	8.88^b^	0.41	< 0.001	< 0.001	0.026
Protein expressions								
Claudin-1/β-actin	1.01^b^	0.38^d^	1.37^a^	0.72^c^	0.06	< 0.001	< 0.001	0.900
ZO-1/β-actin	0.71^b^	0.30^d^	0.98^a^	0.46^c^	0.02	< 0.001	< 0.001	0.018
Occludin//β-actin	0.76^a^	0.23^c^	0.99^a^	0.48^b^	0.08	< 0.001	0.012	0.847
Claudin-3/β-actin	1.33^b^	0.66^c^	1.96^a^	0.99^bc^	0.13	< 0.001	0.006	0.293

^a^Values are means and standard error of the means, *n* = 8 per treatment; labeled means in a row without a common letter differ, *P ≤* 0.05; *CON* Control, *DON* Deoxynivalenol, *DON+RES* Combination of deoxynivalenol and resveratrol, *IL-1β* Interleukin 1 beta, *IL-6* Interleukin 6, *IL-8* Interleukin 8, *RES* Resveratrol, *SEM* Standard error of the mean, *TGF-β* Transforming growth factor beta, *TNF-α* Tumor necrosis factor alpha, *ZO-1* Zonula occludens-1

### Effect of resveratrol supplementation on the redox status in the jejunum of piglets challenged with deoxynivalenol

**Fig. 4:**
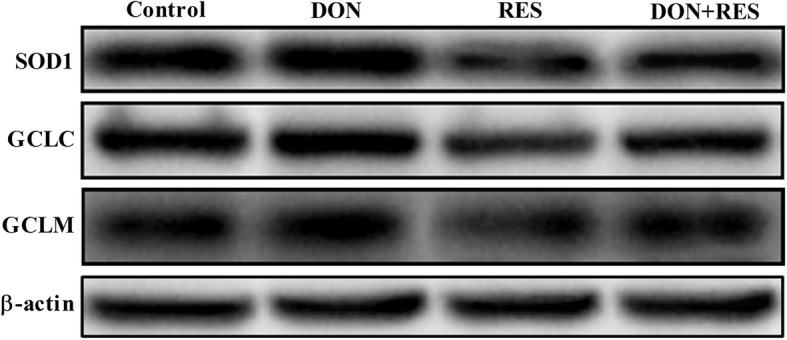
Effect of resveratrol supplementation on the redox status in the jejunum of DON-challenged piglets. Western blot of the amounts of SOD1, GCLM and GCLC protein; loading and transfer of equal amounts of protein was confirmed by the immunodetection of β-actin. Similar results were obtained from 8 independent experiments. DON, deoxynivalenol; DON+RES, combination of deoxynivalenol and resveratrol; GCLC, glutamate-cysteine-ligase catalytic subunit; GCLM, glutamate-cysteine-ligase modulatory subunit; RES, resveratrol; SOD, superoxide dismutase

DON-challenged piglets had lower levels of GSH (*P* = 0.001), T-SOD (*P* = 0.001) and T-AOC (*P* = 0.001) but higher MDA (*P* = 0.001) levels in the jejunum than nonchallenged control piglets (Table 6). Piglets fed diets with RES showed significant increases in the levels of T-SOD (*P* = 0.001) and T-AOC (*P* = 0.001) and decreases in MDA (*P* = 0.001) levels compared with those fed diets without RES (Table 6). A significant DON ×RES interaction was observed for MDA levels (*P* = 0.014); DON-challenged piglets fed a RES-supplemented diet showed significant decreases in MDA levels (Table 6). Additionally, DON exposure markedly inhibited the abundance of *SOD1* (*P* = 0.001)*, GCLC* (*P* = 0.001)*, GCLM* (*P* = 0.001)*,* heme oxygenase-1 (*HO-1*) (*P* = 0.001) and NAD(P) H dehydrogenase, quinone 1 (*NQO-1*) (*P* = 0.001) transcripts in the jejunum compared with the effects of diets without DON (Table 6). RES supplementation significantly increased the mRNA expression levels of *SOD1* (*P* = 0.001)*, GCLC* (*P* = 0.001)*, GCLM* (*P* = 0.001)*, HO-1* (*P* = 0.001) and *NQO-1* (*P* = 0.001) compared with diets without RES (Table 6). A significant DON×RES interaction was observed for the mRNA expression of *SOD1* (*P* = 0.001)*, GCLC* (*P* = 0.001)*, GCLM* (*P* = 0.027)*, HMOX1* (*P* = 0.002) and *NQO-1* (*P* = 0.001); supplementation with RES in DON-challenged piglets prevented the DON-induced decline in *SOD1, GCLC, GCLM, HO-1* and *NQO-1* mRNA expression (Table 6). At the protein level, DON exposure significantly decreased the protein levels of SOD1(*P* = 0.001), GCLC (*P* = 0.001) and GCLM (*P* = 0.001) compared with the effects of diets without DON (Fig. 4 and Table 6). RES supplementation significantly increased the protein levels of SOD1(*P* = 0.010), GCLC (*P* = 0.009) and GCLM (*P* = 0.004) compared with the effects of diets without RES (Fig. 4 and Table 6). A significant DON × RES interaction was observed for the GCLM protein level (*P* = 0.025); supplementation with RES prevented the DON-induced decline in GCLM protein level in DON-challenged piglets (Fig. 4 and Table 6).

**Table 6:** Effect of resveratrol supplementation on the redox status in the jejunum of DON-challenged piglets^a^

Variable	Treatments				SEM	*P*-value		
	CON	DON	RES	DON+RES		DON	RES	RES × DON
Gene expressions involved in antioxidant								
*SOD1*	1.01^b^	0.60^c^	2.52^a^	0.89^b^	0.07	< 0.001	< 0.001	< 0.001
*GCLC*	1.02^b^	0.58^c^	2.47^a^	1.02^b^	0.11	< 0.001	< 0.001	< 0.001
*GCLM*	1.02^b^	0.65^c^	1.80^a^	0.91^bc^	0.12	< 0.001	< 0.001	0.027
*HO-1*	1.01^b^	0.49^c^	2.03^a^	0.97^b^	0.08	< 0.001	< 0.001	0.002
*NQO-1*	1.01^b^	0.58^b^	3.66^a^	1.20^b^	0.25	< 0.001	< 0.001	< 0.001
The contents of antioxidant/oxidant indices, μmol/g protein								
MDA	0.61^b^	0.80^a^	0.53^b^	0.73^a^	0.04	< 0.001	0.041	0.869
T-SOD	63.66^b^	49.95^c^	71.24^a^	57.76^b^	2.36	< 0.001	0.002	0.962
GSH	10.93^ab^	10.02^b^	12.01^a^	11.17^ab^	0.39	0.035	0.008	0.935
T-AOC	0.40^b^	0.29^c^	0.48^a^	0.37^b^	0.03	< 0.001	0.003	0.733
Protein expressions involved in antioxidant								
SOD1/β-actin	1.68^a^	0.39^c^	1.93^a^	0.74^b^	0.10	< 0.001	0.010	0.639
GCLC/β-actin	1.46^b^	0.36^d^	1.81^a^	0.69^c^	0.11	< 0.001	0.009	0.955
GCLM/β-actin	0.79^b^	0.42^c^	1.33^a^	0.52^c^	0.09	< 0.001	0.004	0.025

^a^Values are means and standard error of the means, *n* = 8 per treatment; labeled means in a row without a common letter differ, *P ≤* 0.05; *CON* Control, *DON* Deoxynivalenol, *DON+RES* Combination of deoxynivalenol and resveratrol, *GCLC* Glutamate-cysteine-ligase catalytic subunit, *GCLM* Glutamate-cysteine-ligase modulatory subunit, *GSH* Glutathione, *HO-1* Heme oxygenase-1, *MDA* Malondialdehyde, *NQO-1* NAD(P)H dehydrogenase, quinone 1, *RES* Resveratrol, *SOD* Superoxide dismutase, *SEM* Standard error of the mean, *T-AOC* Total antioxidant capacity

### Effect of resveratrol supplementation on the ultrastructure of intestinal mitochondria in piglets challenged with deoxynivalenol

**Fig. 5:**
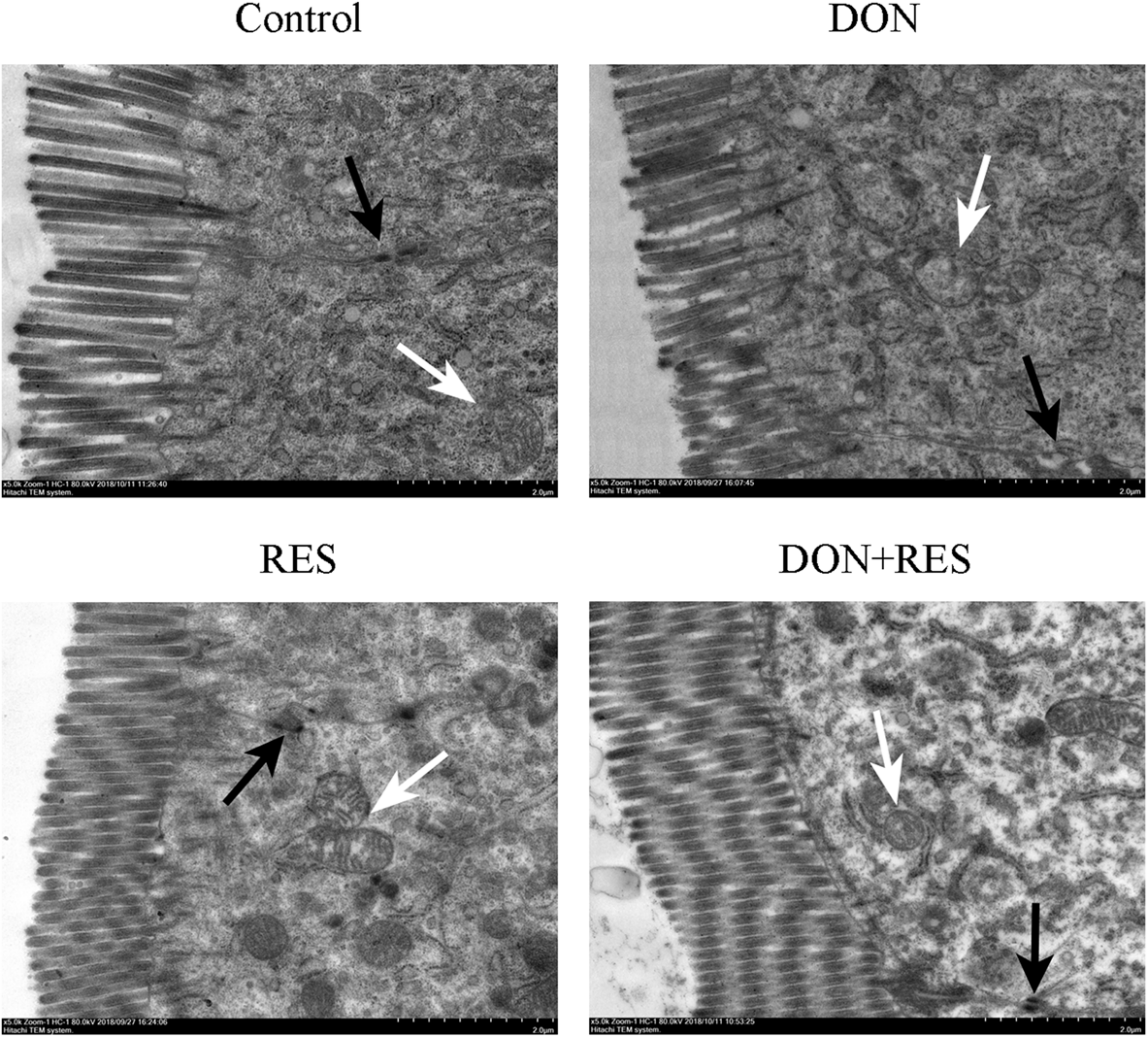
Effect of resveratrol supplementation on the ultrastructure of the intestinal mitochondria in DON-challenged piglets. Piglets’ intestinal sections are shown at 5, 000× magnification, scale bar 2 μm. The white arrows point to the mitochondria. DON, deoxynivalenol; DON+RES, combination of deoxynivalenol and resveratrol; RES, resveratrol

Ultrastructural analysis of mitochondria showed intact membranes and clear cristae mitochondria in nonchallenged piglets. In contrast, fractured and swollen mitochondria with broken and vague cristae were observed in the DON-challenged group (Fig. 5). However, RES supplementation attenuated DON-induced destruction of mitochondria.

### Effect of resveratrol supplementation on the apoptosis of intestinal epithelial cells in piglets challenged with deoxynivalenol

**Fig. 6:**
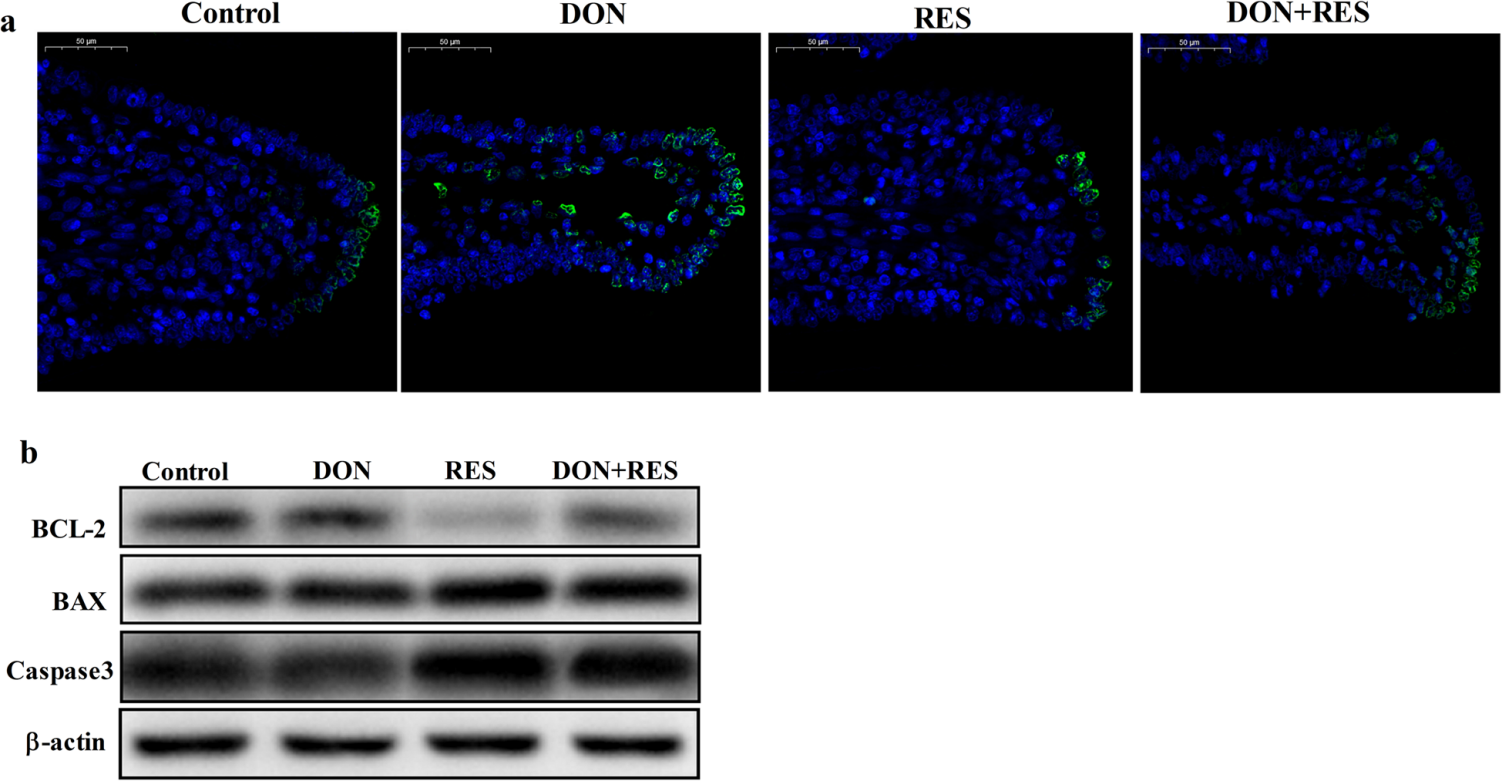
Effect of resveratrol supplementation on the apoptosis of the intestinal epithelial cells in DON-challenged piglets. **a** Immunohistochemical expression for TUNEL in the jejunum of piglets. **b** Western blot of the amounts of BCL-2, BAX and caspase3 protein; loading and transfer of equal amounts of protein was confirmed by the immunodetection of β-actin; similar results were obtained from 8 independent experiments. Original 400× magnification, scale bar 50 μm. Bax, Bcl-2 associated x protein; BCL-2, B-cell lymphoma/leukemia 2; DON, deoxynivalenol; DON+RES, combination of deoxynivalenol and resveratrol; RES, resveratrol; TUNEL, terminal dexynucleotidyl transferase mediated dUTP nick end labeling

The TUNEL assay demonstrated that DON exposure significantly increased DNA fragmentation (*P* = 0.001) in intestinal epithelial cells compared with diets without DON (Fig. 6a and Table 7). RES supplementation significantly reduced DNA fragmentation (*P* = 0.027) compared with diets without RES (Fig. 6a and Table 7). A tendency for a DON × RES interaction for DNA fragmentation (*P* = 0.050) was observed; RES supplementation in DON-challenged piglets prevented the DON-induced increase in DNA fragmentation in intestinal epithelial cells (Fig. 6a and Table 7). Additionally, DON-challenged piglets showed significant increases in the protein levels of BAX (*P* = 0.001) and caspase3 (*P* = 0.001) and a decrease in the protein level of BCL-2 (*P* = 0.001) compared with nonchallenged piglets (Fig. 6b and Table 7). Piglets fed diets supplemented with RES had significantly lower protein levels of caspase3 (*P* = 0.001) than those fed diets without RES (Fig. 6b and Table 7). A significant DON × RES interaction was found for the caspase3 protein level (*P* = 0.001); supplementation with RES prevented the DON-induced an increase in caspase3 protein level in DON-challenged piglets (Fig. 6b and Table 7).

**Table 7:** Effect of resveratrol supplementation on the apoptosis of intestinal epithelial cells in DON-challenged piglets^a^

Variable	Treatments				SEM	*P*-value		
	CON	DON	RES	DON+RES		DON	RES	RES × DON
TUNEL-positive cell percentage								
TUNEL, %	7.44^c^	23.30^a^	6.80^c^	16.16^b^	1.60	< 0.001	0.027	0.050
Protein expressions involved in apoptosis								
BCL-2/β-actin	0.28^a^	0.10^c^	0.29^a^	0.19^b^	0.03	< 0.001	0.095	0.094
BAX/β-actin	0.37^c^	1.12^a^	0.41^c^	0.91^b^	0.07	< 0.001	0.230	0.081
Caspase3/β-actin	0.25^c^	2.09^a^	0.23^c^	0.93^b^	0.11	< 0.001	< 0.001	< 0.001

^a^Values are means and standard error of the means, *n* = 8 per treatment; labeled means in a row without a common letter differ, *P ≤* 0.05; Bax, Bcl-2 associated x protein; *BCL-2* B-cell lymphoma/ leukemia 2, *CON* Control, *DON* Deoxynivalenol, *DON+RES* Combination of deoxynivalenol and resveratrol, *RES* Resveratrol, *SEM* Standard error of the mean, *TUNEL* Terminal deoxynucleotidyl transferase mediated dUTP nick end labeling

### Effects of resveratrol supplementation on the composition of the colonic microbiota and short-chain fatty acid levels of piglets challenged with deoxynivalenol

**Fig. 7:**
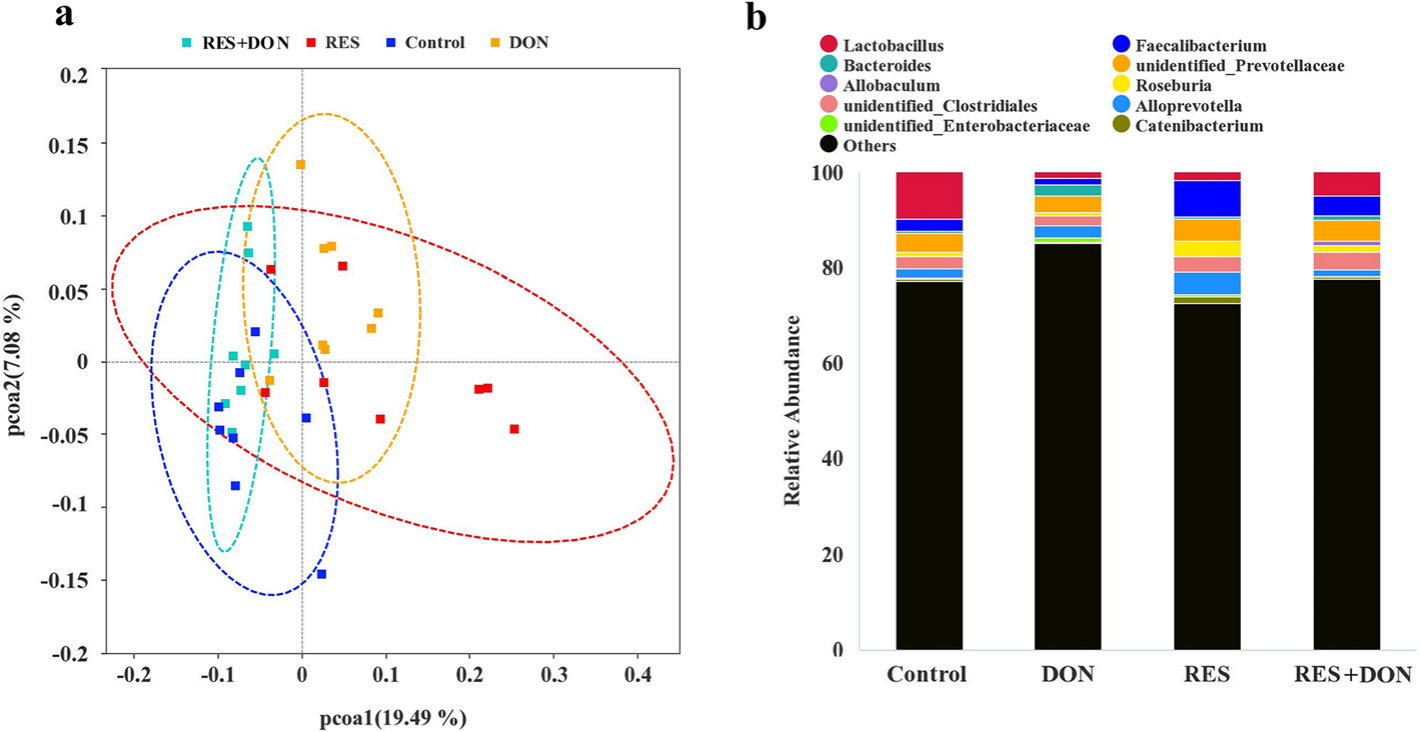
Effects of resveratrol on the gut microbiota of DON-challenged piglets. **a** Principal component analysis of microbial communities in the colon. **b** Stacked bar chart representing the relative abundances of colonic bacteria at the genus level (top 10) in the different dietary groups. DON, deoxynivalenol; DON+RES, combination of deoxynivalenol and resveratrol; RES, resveratrol

As shown in Fig. 7b and Table 8, at the genus level, compared with nonchallenged piglets, DON-challenged piglets showed a significant decrease in the relative abundance of *Roseburia* (*P* = 0.002) and increases in the relative abundances of *Bacteroides* (*P* = 0.001) and *unidentified-Enterobacteriaceae* (*P* = 0.001). Supplementation with RES significantly increased the abundances of *Faecalibacterium* (*P* = 0.001) and *Roseburia* (*P* = 0.001), while decreasing the abundances of *Bacteroides* (*P* = 0.001) and *unidentified-Enterobacteriaceae* (*P* = 0.001) compared with diets without RES. A significant DON ×RES interaction was observed for the abundances of *Lactobacillus* (*P* = 0.005), *Roseburia* (*P* = 0.048), *Bacteroides* (*P* = 0.001) and *unidentified-Enterobacteriaceae* (*P* = 0.001); RES supplementation in DON-challenged piglets prevented the DON-induced decline in the relative abundance of *Roseburia,* and the increases in the relative abundances of *Bacteroides* and *unidentified-Enterobacteriaceae*; the relative abundance of *Lactobacillus* was increased in DON-challenged piglets fed a RES-supplemented diet compared with that in DON-challenged piglets or piglets fed a diet with RES alone.

**Table 8:** Effects of resveratrol on short-chain fatty acid and gut microbiota in the colon digesta of weaned piglets challenged with deoxynivalenol^a^

Variable	Treatments				SEM	*P*-value		
	CON	DON	RES	DON+ RES		DON	RES	DON×RES
Relative abundance of total sequences at the genus level, %								
*Lactobacillus*	9.77^a^	1.05^b^	1.57^b^	4.79^ab^	1.96	0.266	0.172	0.005
*Faecalibacterium*	2.49	1.47	7.60	4.18	1.09	0.489	0.001	0.285
*Bacteroides*	0.42^c^	2.25^a^	0.66^c^	0.42^b^	0.17	0.001	0.001	0.001
*Roseburia*	0.90^b^	0.42^c^	3.29^a^	1.35^b^	0.37	0.002	0.001	0.048
*Unidentified-Enterobacteriaceae*	0.12^b^	1.05^a^	0.13^b^	0.07^b^	0.05	0.001	0.001	0.001
SCFAs concentrations in the colon digesta, μg/g digesta								
Acetate	194.18	204.19	190.72	192.28	12.14	0.638	0.531	0.731
Propionate	151.51	150.65	150.14	145.12	10.49	0.785	0.749	0.847
Butyrate	105.56^ab^	70.52^c^	110.91^a^	95.75^b^	4.93	<0.001	0.003	0.046

^a^Values are means and standard error of the means, *n* = 8 per treatment; labeled means in a row without a common letter differ, *P ≤* 0.05; *CON* Control, *DON* Deoxynivalenol, *DON+RES* Combination of deoxynivalenol and resveratrol, *RES* Resveratrol, *SCFA* Short-chain fatty acid, *SEM* Standard error of the mean

Compared with nonchallenged piglets, DON-challenged piglets showed a significant decrease in butyrate level (*P*<0.001) (Table 8). RES supplementation significantly increased butyrate levels (*P =* 0.003) compared with diets without RES (Table 8). A significant DON ×RES interaction was observed for butyrate levels (*P* = 0.046); RES supplementation in DON-challenged piglets reversed the DON-induced decline in the level of butyrate (Table 8). Additionally, no treatment effects were observed for the levels of acetate and propionate.

### Correlation analysis of the gut microbiota and variables related to intestinal barrier function, inflammation and oxidative damage

**Fig. 8:**
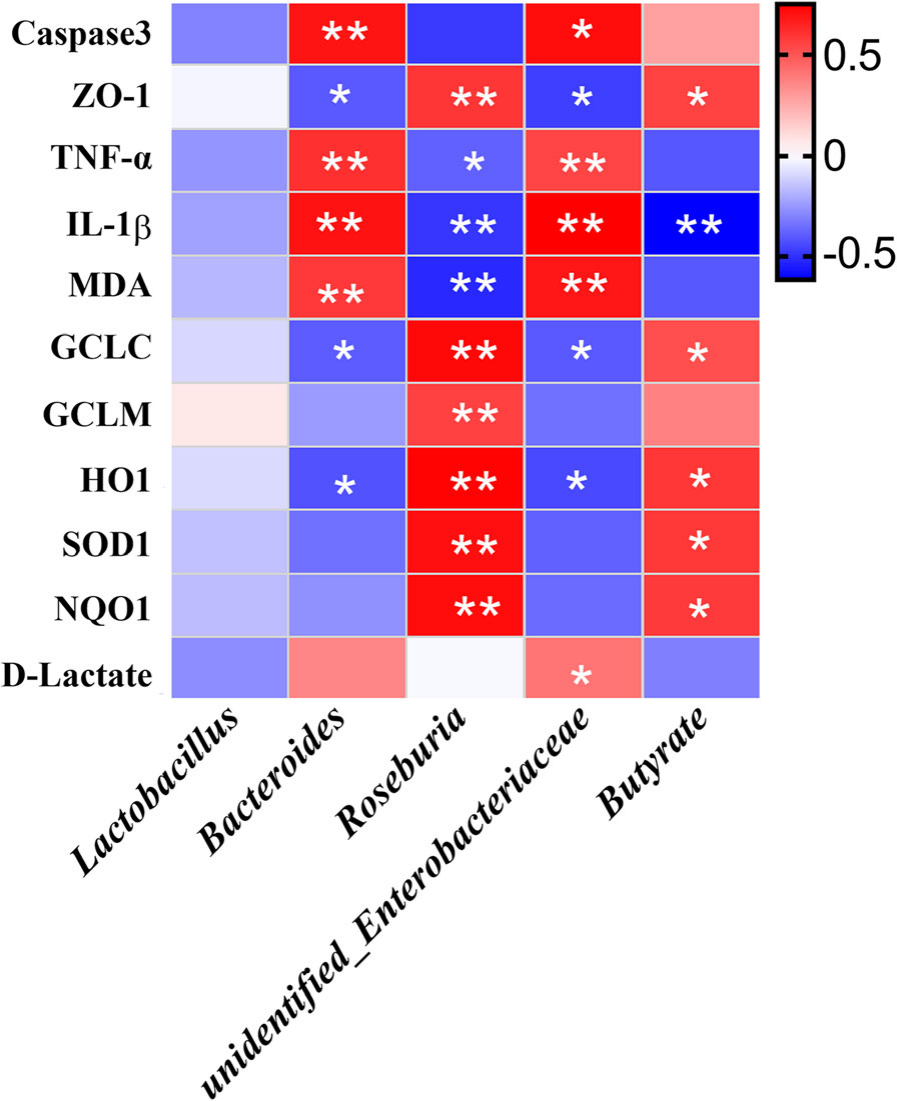
Heatmap of Pearson’s correlation coefficients between the relative abundances of genera and variables related to intestinal barrier function, inflammation and oxidative damage affected by deoxynivalenol and resveratrol. In the panel, * *P ≤* 0.05 and ** *P* < 0.01. Red with a *P ≤* 0.05 represents a significant positive correlation, blue with a *P ≤* 0.05 represents a significant negative correlation, and white represents no correlation. IL-1β, interlleukin 1 beta; GCLC, glutamate-cysteine-ligase catalytic subunit; GCLM, glutamate-cysteine-ligase modulatory subunit; HO-1, heme oxygenase-1; MDA, malondialdehyde; NQO-1, NAD(P) H dehydrogenase, quinone 1; TNF-α, tumor necrosis factor α; ZO-1, zonula occludens-1

A Pearson correlation analysis was used to investigate the correlations between variables related to intestinal barrier function, inflammation and oxidative damage and the abundances of the main microbial genera (Fig. 8). The relative abundance of *Roseburia* showed positive correlations with the mRNA expression of *ZO-1, GCLC, GCLM, HO-1, SOD1,* and *NQO1* but was negatively associated with the mRNA expression of *TNF-α* and *IL-1β* and the MDA levels in the jejunum. The abundance of *Bacteroides* was positively correlated with caspase-3 protein expression, the mRNA expression of *TNF-α* and *IL-1β*, and MDA levels but showed a negative correlation with the mRNA expression of *ZO-1, GCLC* and *HO-1*. The abundance of *unidentified- Enterobacteriaceae* was positively correlated with the protein expression of caspase3, the MDA levels, mRNA expression of *TNF-α* and *IL-1β* in the jejunum, and plasma D-lactate concentrations and showed a negative correlation with *ZO-1, GCLC* and *HO-1* mRNA expression. Butyrate concentrations showed positive correlations with the mRNA expression of *ZO-1, GCLC, HO-1, SOD1,* and *NQO1* but were negatively associated with the mRNA expression of *IL-1β* in the jejunum.

## Discussion

Increasing evidence indicates that the beneficial effects of RES include improving intestinal barrier function and reducing oxidative damage and inflammation [32, 33]. Thus, we investigated the protective effect of RES on intestinal barrier function and the inflammatory response in DON-challenged piglets. As expected, our results showed that RES supplementation improved BW gain, ADG and gain/feed, which was in accordance with the findings of previous polyphenol-investigated studies [13, 23, 34]. Additionally, we observed that DON significantly impaired the growth performance of piglets, which was consistent with the results of previous studies [33, 35, 36]. However, RES supplementation did not significantly prevent the DON-induced reduction in growth performance. These findings indicated that the digestive and absorptive functions of RES may be compromised during DON exposure. Until now, studies on RES supplementation-mediated modulation of the growth performance of DON-challenged piglets have been limited.

Villus height, crypt depth, and VCR are useful criteria for evaluating intestinal health and functions [37]. In the present study, DON exposure decreased jejunal villus height and jejunal VCR, suggesting that DON caused acute intestinal mucosal damage. Our findings were similar to the results of Wang et al. [38]. Several previous studies found that polyphenols caused increases in villus height and VCR in pigs [34, 39]. Consistent with these findings, independent of DON challenge, RES supplementation increased jejunal villus height and VCR relative to the diets without RES in the present study, indicating that RES improved intestinal morphology. Similar to our results, a study by Zhang et al. reported that a diet enriched in RES attenuated radiation-induced intestinal injury [40].

Several indices, such as DAO and D-lactate activity, are commonly used to assess intestinal barrier function [41, 42]. Some recent studies have demonstrated that polyphenols, such as tannic acid, RES and pterostilbene significantly diminish plasma DAO and D*-*lactate concentrations [12, 43]. Consistent with these observations, our results found that supplementation with RES significantly reduced the levels of plasma DAO and D-lactate in weaned piglets. Meanwhile, RES significantly suppressed the release of plasma D-lactate in DON-challenged piglets. One potential explanation for this finding could be the RES-mediated upregulation of *ZO-1* mRNA and protein in the jejunum of DON-challenged piglets. ZO-1 is a major integral membrane protein that can limit epithelial permeability to low molecular mass molecules and maintain the barrier function of the small intestine [44]. Similarly, a previous study demonstrated that RES protected against H_2_O_2_-induced intestinal epithelial barrier dysfunction by increasing the expression of ZO-1 and occludin in weaned piglets [45]. Collectively, these results suggested that RES supplementation could improve intestinal integrity in DON-challenged piglets.

Many studies have reported that DON can induce inflammation *in vivo* and *in vitro* [9, 11, 30, 38]. Meanwhile, a large number of studies with either porcine intestinal epithelial cell culture or experimental pig models of inflammation have convincingly demonstrated that polyphenols are capable of suppressing experimentally induced inflammation [11, 12, 16, 17, 46]. Thus, we hypothesized that RES supplementation also exerts beneficial effects on the intestine by alleviating the DON-induced intestinal inflammatory response. A previous study reported that RES supplementation prevented TNF-α production in piglets infected with rotavirus [47]. Consistent with this observation, the results in the present study showed that dietary RES supplementation reversed the DON-induced increases in the mRNA and protein expression of TNF-α and IL-1β, suggesting that providing DON-challenged piglets with diets containing RES can efficiently inhibit intestinal inflammation.

The intestine is susceptible to oxidative damage, particularly under stressful conditions [48]. Our previous study determined that DON exposure increased oxidative stress and induced apoptosis in IPEC-J2 cells [9, 11]. Similarly, a study by Wu et al. showed that DON increased MDA concentrations in the blood and small intestine, which is a sensitive indicator of oxidative damage [49]. Consistent with these observations, our study showed that DON-challenged piglets exhibited elevated MDA levels and decreased GSH, T-SOD and T-AOC levels in the jejunum. Polyphenols have been shown to exert antioxidative effects in intestinal epithelial cell culture and weaned piglet studies [13–15, 34, 39]. Consistent with these findings, the results in the present study showed that supplementation with RES suppressed MDA levels while increasing T-SOD and T-AOC contents. Additionally, RES supplementation inhibited MDA levels in DON-challenged piglets. Similar to our finding, a study by Cao et al. indicated that dietary RES attenuated oxidative stress in piglets treated with diquat, as indicated by lower MDA levels in the jejunal mucosa [21]. As expected, aside from its antioxidant effect, RES also promoted increases in antioxidant enzymes and antioxidant genes in the jejunum of DON-challenged piglets, as indicated by increased levels of *GCLC, GCLM, HO-1, NQO1* and *SOD1* and enhanced protein expression of GCLM. GCLC, GCLM, HO-1, NQO1 and SOD1 are downstream targets of NRF2, which plays an important role in the antioxidant response [11, 50, 51]. Consistent with the present findings, our previous study showed that the mRNA levels of *SOD1, GCLC* and *GCLM* in IPEC-J2 cells were suppressed by DON, and pretreatment with RES blocked these effects [11].

Inflammation and oxidative damage usually promote small intestinal epithelial cell apoptosis [52, 53]. The BCL-2/BAX/caspase3 signaling pathway has been reported to regulate cell apoptosis [54]. In the present study, we found that DON challenge induced intestinal epithelial cell apoptosis, which was characterized by increases in the propotion of TUNEL-positive cells and the protein expression of BAX and caspase3 and a reduction in BCL2 expression. Similarly, previous studies have demonstrated that DON can induce apoptosis in enterocytes and leukomonocytes through the upregulation of BAX and caspase3 and downregulation of BCL-2 [55, 56]. However, RES supplementation alleviated DON-induced apoptosis, as indicated by reductions in the rate of TUNEL-positive cells and the protein expression of caspase3 in DON-challenged piglets fed the RES-supplemented diet. These findings were further supported by the analysis of mitochondrial ultrastructure, which showed that RES supplementation could reverse the DON-induced membrane damage, broken cristae, vacuolization and turgidity of mitochondria. Consistent with the present results, our previous study showed that RES could efficiently attenuate IPEC-J2 cell apoptosis by activating NRF2 signaling [11]. Cao et al. also demonstrated that dietary RES improved diquat-induced ultrastructural dysfunction of mitochondria in piglets [21]. Collectively, these results indicated that RES supplementation effectively protected against DON-induced intestinal oxidative damage and apoptosis.

The intestinal microbiota represents a crucial bridge between environmental substances and host health. In the present study, increased relative abundances of *Bacteroides* and *unidentified Enterobacteriaceae* and decreased relative abundance of *Roseburia* were observed in DON-exposed piglets. However, RES supplementation increased the abundance of *Roseburia* and decreased the abundances of *Bacteroides* and *unidentified- Enterobacteriaceae* in DON-challenged piglets. A previous study showed that maternal dietary RES increased the abundance of butyrate-producing bacteria, such as *Flavonifractor*, *Odoribacter* and *Oscillibacter* in weaning piglets [12]. However, until now, limited data on the direct effect of DON on the intestinal microbiota of piglets have been published, and a discrepancy exists among those studies [18, 19]. Enrichment of *Bacteroides* and *unidentified-Enterobacteriaceae* has been shown to be involved in mucosal inflammation, which can trigger colitis upon disruption of the barrier function of colonic epithelial cells [57, 58]. *Roseburia,* which is the main butyrate-producing bacteria in the colon, has been repeatedly reported to be crucial for the prevention of pathogen infection and alleviation of intestinal inflammation [59]. Therefore, the increasing *Roseburia* abundance by RES in DON-challenged piglets may result in increased butyrate production. Butyrate is one of the most prominent short-chain fatty acid (SCFA), which are produced by gut microbiota fermentation of nondigestible carbohydrates [60]. Previous studies have reported that butyrate stimulates the growth and differentiation of enterocytes, exerts antioxidant and anti-inflammatory effects and modulates intestinal barrier function [61–63]. In the present study, supplementation with RES reversed the DON-induced decline in the butyrate level in the colonic digesta, which was supported by the increased abundance of *Roseburia*. Altogether, based on these results, we conclude that RES-mediated alleviation of the DON-induced inflammatory response and oxidative damage may be linked to changes in the gut microbiota and butyrate levels. However, further study is warranted to explore the contribution of the gut microbiome to DON-induced intestinal inflammation and oxidative damage, and thus a fecal microbiota transplantation experiment, in which DON-challenged piglets are colonized with intestinal microbiota from control or RES-treated piglets should be carried out.

Limitation to the study was the use of zinc oxide at a pharmacological level (ZnO, 2,000 ppm) as an alternative to antibiotics in the experiments conducted to prevent postweaning diarrhea, as ZnO may elicit direct effects on host pathways, distort the effects of DON and RES on the microbial composition and may prove difficult to replicate in future studies. Notably, all piglets in these experiments were supplemented with the same ZnO regimen, and thus the differences observed should reflect the dietary effects (DON versus RES versus interactive effects of DON and RES).

## Conclusions

Collectively, the results of the present study indicated that RES supplementation may efficiently alleviate intestinal inflammation and oxidative damage and thereby improve the intestinal integrity of DON-challenged piglets. The protective effects of RES on gut health may be closely related to increased *Roseburia* abundance and butyrate levels, and decreased abundances of *Bacteroides* and *unidentified-Enterobacteriaceae*.

## Data Availability

The datasets analyzed in the current study are available from the corresponding author on reasonable request.

## Abbreviations

Bax: Bcl-2 associated x protein
BCL-2: B-cell lymphoma / leukemia 2
DAO: Diamine oxidase
DON: Deoxynivalenol
DON+RES: Combination of deoxynivalenol and resveratrol
GCLC: Glutamate-cysteine-ligase catalytic subunit
GCLM: Glutamate-cysteine-ligase modulatory subunit
GSH: Glutathione
HO-1: Heme oxygenase-1
IL-1β: Interlleukin 1 beta
IL-6: Interlleukin 6
IL-8: Interlleukin 8
MDA: Malondialdehyde
NQO-1: NAD(P) H dehydrogenase, quinone 1
RES: Resveratrol
SOD: Superoxide dismutase
T-AOC: Total antioxidant capacity
TGF-β: Transforming growth factor β
TNF-α: Tumor necrosis factor alpha
TUNEL: Terminal dexynucleotidyl transferase mediated dUTP nick end labeling
VCR: Villi height to crypt depth
ZO-1: Zonula occludens-1
